# Editorial Department

**Published:** 1856-02

**Authors:** 


At Chicago, where they have a new building for the Medical College, Prof. Brainerd, in his introductory, gives a history of the school, and thus alludes to the gratuitous plan of education:

"There may have been those among us, who allowed themselves for a moment to hope that placing Medical Schools under the patronage of the State, and making the institution free, while the courses were made more thorough, and the requirements for graduation much greater, would be the means of effecting that improvement in the profession, admitted on all hands to be so desirable. I confess myself to have been of the number who favored this view, but I have not the pretension to have learned nothing by experience, and to have always retained the same opinion. On the contrary the events of the last few years have been full of instruetion for me, and the trial which hae been made of this system in the State of Michigan, has convinced me that, however imperfect medical schools may be, as commonly organized, those supported by the State, and controlled by public officers will not be less so, and that no improvement is to be expected from the State governments who are themselves unenlightened on the subject of medical instruction."

